# Tranexamic acid decreases blood loss in shoulder arthroplasty: A meta-analysis

**DOI:** 10.1097/MD.0000000000007762

**Published:** 2017-08-18

**Authors:** Bin-feng Yu, Guo-jing Yang, Qi Li, Liang-le Liu

**Affiliations:** Department of Orthopedics, The Third Affiliated Hospital of Wenzhou Medical College, Wenzhou, Zhejiang Province, People's Republic of China.

**Keywords:** arthroplasty, blood loss, meta-analysis, shoulder, tranexamic acid

## Abstract

**Background:**

The objective of this meta-analysis was to evaluate the efficacy and safety of tranexamic acid (TXA) in shoulder arthroplasty (SA).

**Methods:**

Academic articles were identified from the Cochrane Library, Medline (1966–2017.2), PubMed (1966–2017.2), Embase (1980–2017.2), and ScienceDirect (1966–2017.2). Randomized controlled trials (RCTs) and non-RCTs studying TXA in SA were included. Two independent reviewers conducted independent data abstraction. The *I*^2^ statistic was used to assess heterogeneity. Fixed- or random-effects models were used for meta-analysis.

**Results:**

Two RCTs and 2 non-RCTs met the inclusion criteria. This meta-analysis found significant differences in postoperative hemoglobin reduction (MD = –0.71 g/dL), drainage volume (MD = –133.21 mL), and total blood loss (MD = –226.82 mL) between TXA groups and controls. There were no significant differences in blood transfusion requirements, operation time, or length of hospital stay.

**Conclusions:**

The use of TXA in SA decreases postoperative hemoglobin reduction, drainage volume, and total blood loss and does not increase the risk of complications. Because of the limited high-quality evidence currently available, additional randomized controlled trials are required.

## 1 Introduction

Shoulder arthroplasty (SA) is an effective method to relieve pain and restore joint function in patients with severe shoulder disease.^[1]^ Unfortunately, SA is particularly prone to large volumes of blood loss.^[2]^ Studies have reported that the rates of blood transfusion after total shoulder arthroplasty (TSA) range from 7.4% to 43%.^[3,4]^ Patients undergoing reverse total shoulder arthroplasty (RTSA) are at even higher risk of requiring a postoperative blood transfusion.^[5]^ Allogenic blood transfusion is associated with risks including transmission of viruses, immunologically mediated disease, and cardiovascular dysfunction, which can result in financial burdens and patient morbidity and mortality.^[6–8]^

Tranexamic acid (TXA) is a popular antifibrinolytic agent that has been gaining popularity for use in the following various surgical procedures.^[9]^ A number of studies have found that the use of TXA reduced perioperative blood loss and the need for blood transfusions following total knee arthroplasty (TKA) and total hip arthroplasty (THA) without increasing the risk for venous thromboembolism (VTE).^[10–12]^ To date, few studies have examined the use of TXA in shoulder arthroplasty^[13–16]^; however, their results are not consistent. Moreover, the existing studies have been plagued by limitations, including small samples, inconclusive results, and inaccurate evaluations. Therefore, the current study was conducted to critically review and summarize the literature to assess the safety and efficacy of TXA in SA.

## 2 Material and methods

### 2.1 Search strategy

**Figure 1:**
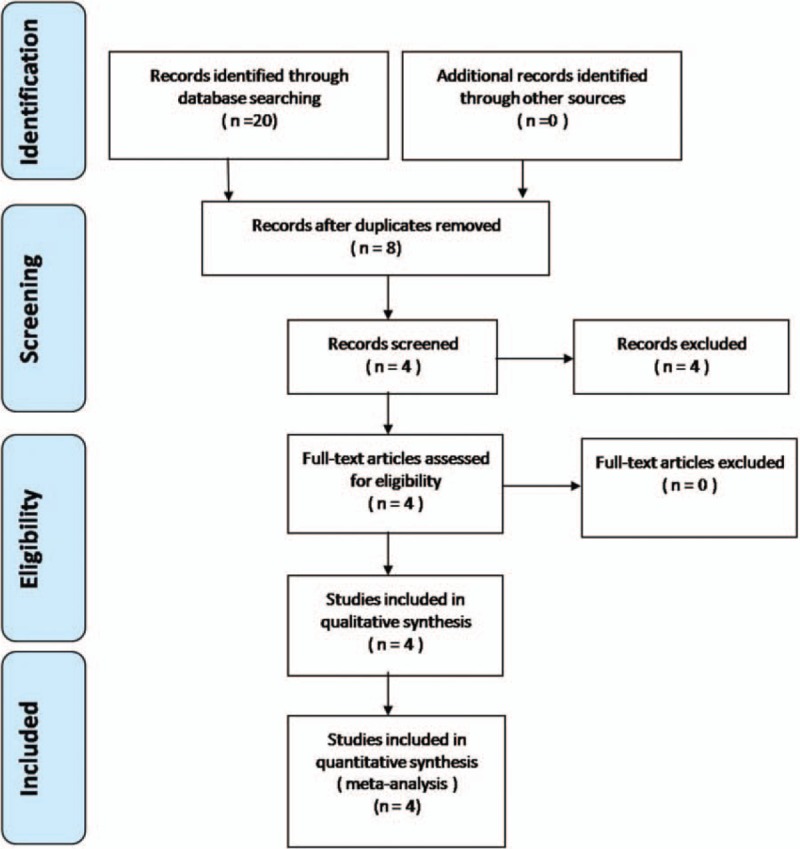
Flowchart of the study selection process.

Electronic databases such as Cochrane Library, Medline (1966–2017.2), PubMed (1966–2017.2), Embase (1980–2017.2), and ScienceDirect (1985–2017.2) were searched. We then manually searched the reference lists of all included studies, relevant books, review articles, and meeting proceedings to identify trials that might have been missed in the initial electronic search. The search strategy is illustrated in Fig. 1. The key words “shoulder,” “replacement or arthroplasty,” and “tranexamic acid” were used in combination with the Boolean operators AND or OR. Because this was a meta-analysis, no ethics committee or institutional review board approval was required.

### 2.2 Inclusion criteria

Studies were considered eligible for inclusion if they met the following criteria: (1) the patients underwent primary SA; (2) the intervention was the use of tranexamic acid, with the use of a placebo (control) group; (3) the outcomes included blood loss, blood transfusion, hemoglobin reduction, clinical outcomes, and complications; and (4) the study was a published or unpublished comparative trial.

### 2.3 Exclusion criteria

We excluded studies as follows: (1) those without a control group; (2) studies with no available full-text version; (3) studies with no available outcome data; and (4) studies of revision SA.

### 2.4 Selection criteria

Two reviewers independently screened the titles and abstracts according to the eligibility criteria. The full text of the studies that potentially met the inclusion criteria were subsequently read, and the literature was reviewed to determine suitability of final inclusion. Disagreement was resolved by consulting with a third reviewer.

### 2.5 Quality assessment

Depending on whether a study was a randomized or nonrandomized trial, the Methodological Index for Non-randomized Studies (MINORS) form was used to assess retrospective controlled trials.^[17]^ Quality assessment for RCT was conducted according to a modification of the generic evaluation tool used by the Cochrane Bone, Joint and Muscle Trauma Group.^[18]^ To determine the risk of bias, quality criteria included (i) details of randomization method, (ii) allocation concealment, (iii) blinding of participants and personnel, (iv) blind outcome assessment, (v) incomplete outcome data, (vi) selective outcome reporting, and (vii) other sources of bias. Disagreements were resolved by consensus or consultation with the senior reviewer.

### 2.6 Data extraction

Two researchers independently extracted the data from the included literature. In the case of incomplete data, the corresponding author was consulted for details. The following information was extracted: first author name, year of publication, intervening measures, comparable baseline, sample size, and outcome measures. Other relevant parameters were also extracted from individual studies.

### 2.7 Data analysis and statistical methods

Pooling of data was analyzed using RevMan 5.1 (The Cochrane Collaboration, Oxford, United Kingdom). Heterogeneity was estimated depending on the values of *P* and *I*^2^ using the standard chi-square test. When *I*^2^ > 50%, *P* < .1 was considered to be significant heterogeneity. Therefore, a random-effects model was applied for data analysis. A fixed-effects model was used when no significant heterogeneity was found. In the case of significant heterogeneity, subgroup analysis was performed to investigate its sources. For continuous outcomes, the mean difference (MD) and 95% confidence interval (CI) were presented. Risk difference (RD) and 95% CIs were calculated for dichotomous data.

## 3 Results

### 3.1 Search results

A total of 20 studies were identified as potentially relevant literature reports. After titles and abstracts were scanned, 16 reports were excluded according to the eligibility criteria. No additional studies were obtained after the reference review. Ultimately, 2 RCTs and 2 non-RCTs were eligible for data extraction and meta-analysis. The search process is shown in Fig. 1.

### 3.2 Risk of bias assessment

**Figure 2:**
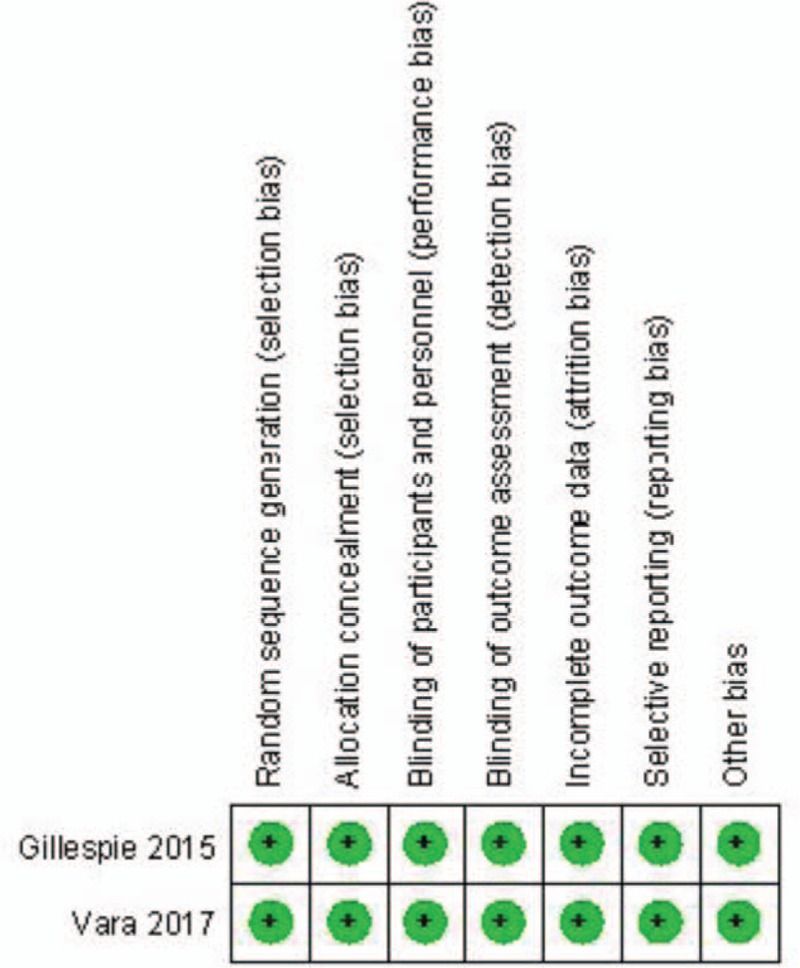
Summary of bias risk of randomized controlled trials.

The RCT quality was assessed based on the Cochrane Handbook for Systematic Review of Interventions (Fig. 2). Both RCTs stated clear inclusion and exclusion criteria and included adequate methodology of randomization, concealment of allocation, blinding, and intent-to-treatment analysis. No unclear bias due to incomplete outcome data or selective outcomes was reported. For the non-RCTs, the MINORS scores were 17 to 18 for the retrospectively controlled trials. The methodological quality assessment is illustrated in Table 1.

**Table 1:**
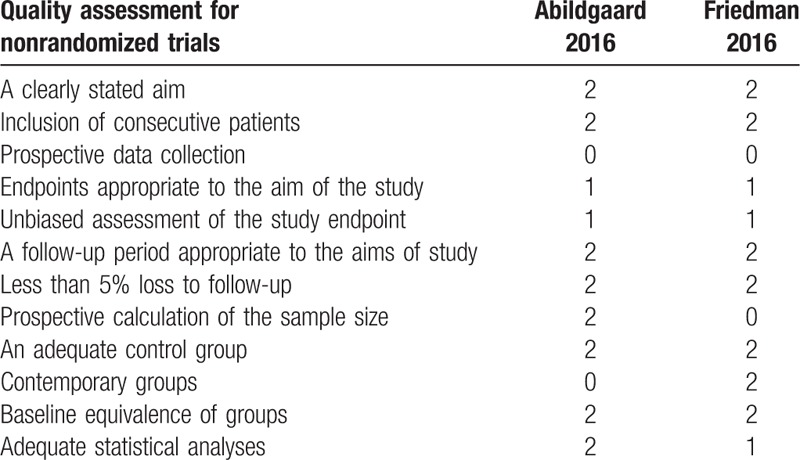
Quality assessment score of the retrospective studies.

### 3.3 Study characteristics

Demographic characteristics and details concerning the literature type of the included studies are summarized in Table 2. Statistically similar baseline characteristics were observed in both groups. All studies had small sample sizes between 102 and 171 shoulders.

**Table 2:**
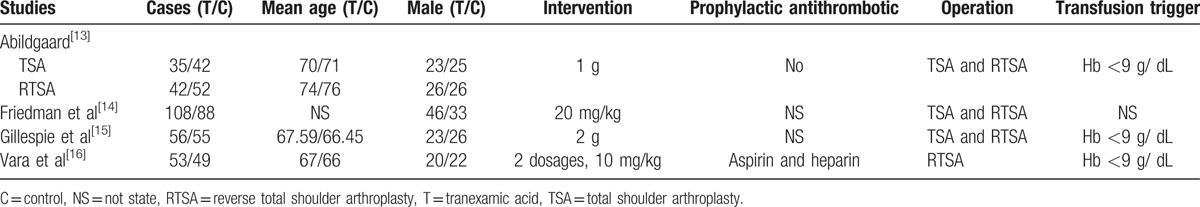
Cohort characteristics.

### 3.4 Outcomes of meta-analysis

#### 3.4.1 Total blood loss

**Figure 3:**
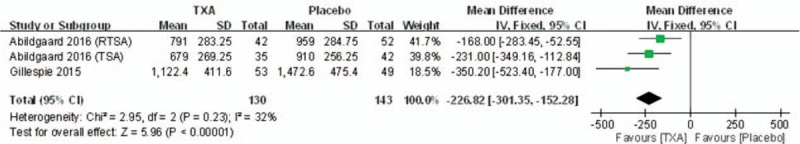
Forest plot diagram showing total blood loss between 2 groups.

Total blood loss was reported in 4 studies. No significant heterogeneity was found using the fixed-effects model (*I*^2^ = 32%, *P* = .23). Total blood loss in the TXA group was significantly lower than that in the control group (MD = –226.81, 95% CI: –301.35 to –152.28, *P* < .00001; Fig. 3).

#### 3.4.2 Drainage volume

**Figure 4:**
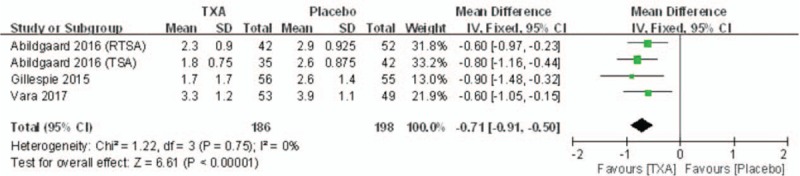
Forest plot diagram showing drain volume between 2 groups.

Drainage volume was reported in 3 studies. Significant heterogeneity was found when a random-effects model was applied (*I*^2^ = 82%, *P* = .0001). The drainage volume in TXA groups was significantly lower than that in control groups (MD = –133.21, 95% CI: –194.66 to –71.77, *P* < .0001; Fig. 4).

#### 3.4.3 Hemoglobin reduction

**Figure 5:**
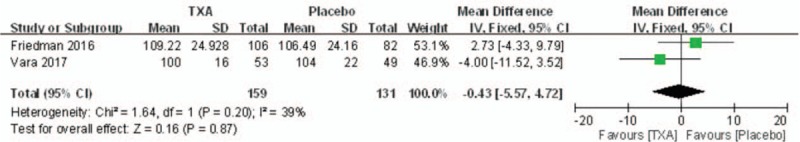
Forest plot diagram showing postoperative hemoglobin reduction between 2 groups.

Hemoglobin reduction was reported in 3 of the studies. No significant heterogeneity was found when a fixed-effects model was applied (*I*^2^ = 0%, *P* = .75). Hemoglobin reduction in TXA groups was significantly lower than that in control groups (MD = –0.71, 95% CI: –0.91 to –0.50, *P* < .00001; Fig. 5).

#### 3.4.4 Rate of blood transfusion

**Figure 6:**
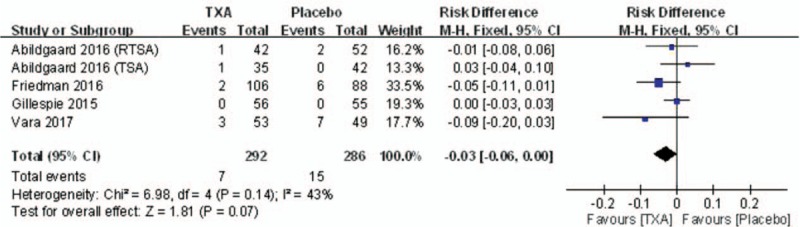
Forest plot diagram showing the blood transfusion rate between 2 groups.

Four studies reported the rate of blood transfusion following SA. There was no significant heterogeneity (*I*^2^ = 43%, *P* = .14); therefore, a fixed-effects model was utilized. Pooling results demonstrated that the rate of blood transfusion in the TXA group was not significantly lower than that in the control group (RD = –0.03, 95% CI: –0.06 to 0.00, *P* = .07; Fig. 6).

#### 3.4.5 Length of surgery

**Figure 7:**
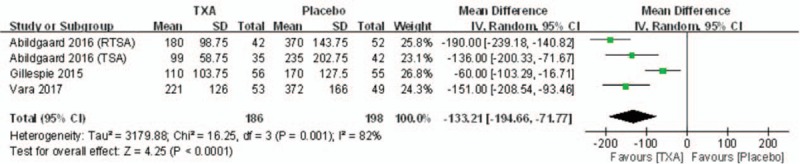
Forest plot diagram showing the length of surgery between 2 groups.

Length of surgery was reported in 2 studies. No significant heterogeneity was found using a fixed-effects model (*I*^2^ = 39%, *P* = .20). There was no significant difference between the 2 groups regarding the length of surgery (MD = –0.43, 95% CI: –5.57 to 4.72, *P* = .87; Fig. 7).

#### 3.4.6 Hospital stay

**Figure 8:**
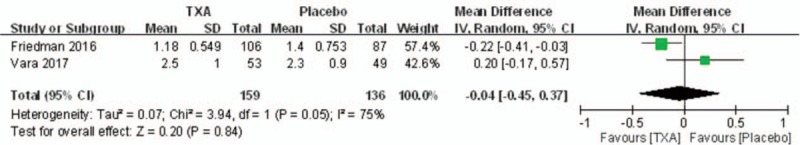
Forest plot diagram showing hospital stay between 2 groups.

Two articles reported on the length of hospital stay. A random-effects model was used due to the significant heterogeneity (*I*^2^ = 75%, *P* = .05). No significance difference was observed between groups regarding hospital stay (MD = –0.04, 95% CI: –0.45 to 0.37, *P* = .84; Fig. 8).

## 4 Discussion

To our knowledge, this is the first meta-analysis to assess the efficiency of TXA in primary SA. The most significant findings of this meta-analysis were that TXA in primary SA reduces postoperative hemoglobin reduction, total blood loss, and drainage volume. Furthermore, no TXA-related adverse effects were discovered.

Two RCTs and 2 non-RCTs were included in the current meta-analysis. Some methodological weaknesses existed in the included studies, which influenced the strength of the point estimates. Both RCTs were of high methodological quality. The shortcomings of the 2 non-RCTs weakened the level of evidence. Although we searched the electronic database systematically, language bias and publication bias may have caused some reports to have been omitted. Furthermore, the sample size is relatively small in the literature as a whole.

TXA, an antifibrinolytic agent, is a synthetic derivative of the amino acid lysine. The mechanisms of TXA action are to decrease the physiologic process of fibrinolysis and to prevent the degradation of fibrin.^[19]^ Moreover, TXA has an anti-plasmin effect and may inhibit the platelet-activating factor, whereby it may protect platelets. Blood loss following SA is a major concern that affects functional outcome and long-term prognosis.^[20–22]^ In our meta-analysis, pooled results showed that TXA would reduce drainage volume (MD = –133.21 mL) and total blood loss (MD = –226.81 mL) in SA. These results are similar to those reported in the literature for TKA and THA.^[19,23]^

Blood transfusions can lead to severe complications and increase medical costs. Therefore, the importance of blood management in the SA procedure is essential. This meta-analysis showed that TXA decreased postoperative hemoglobin reduction (MD = –0.71 g/dL) without reducing the rate of blood transfusion (RD = –0.03) in SA. Although the blood transfusion rate was lower in the TXA group, there was no significant difference between the 2 groups (7/292 vs 15/286). This may have been due to variations in blood transfusion trigger criteria. One of included studies did not mention clear transfusion triggers.^[14]^ Furthermore, the study's sample size was too small to determine whether TXA reduces the need for transfusion in SA. TXA has been shown to reduce the need for transfusions by one-third in a meta-analysis of THA and TKA.^[19,23]^

Friedman et al^[14]^ reported that TXA reduced recovery room time and hospitalization time in SA. In TKA, the hospital stay for those treated with TXA was on average 24% shorter than that for patients who did not receive TXA.^[24]^ The current meta-analysis, however, did not find that TXA shortened hospital stay in SA. This may have been because patients with SA usually experience earlier postoperative mobilization than those undergoing TKA.

TXA is a well-tolerated drug, with its most commonly reported side effects limited to nausea and diarrhea.^[23]^

The included studies did not report any postoperative complications or side effects. Therefore, we do not have enough evidence to confirm whether TXA increases the risk of complications. Previous studies found no significant difference in DVT, PE, infection rates, or other complications.^[25,26]^

Several factors, including dosage of TXA, prosthesis design, timing of intravenous administration, and surgical techniques, influence the efficacy and safety of TXA. Jiang et al^[27]^ found that RTSA patients had significantly higher deep venous thrombosis and blood transfusion rates compared with TSA patients. A meta-analysis was conducted by Zhou et al, who found that intravenous administration of 10 to 20 mg/kg (or 1 g) of TXA preoperatively, with or without 10 to 20 mg/kg 3–12 hours postoperatively, appears to be safe and effective in THA.^[19]^ The TXA dosage of the studies included in this meta-analysis was comparable.

Several potential limitations should be noted. (1) Only 4 studies were included, and the sample size of each was relatively small. (2) Some outcome parameters such as function score and range of motion were not fully described and were, therefore, not subject to meta-analysis. (3) Because of the small samples of included studies, subgroup analysis was not performed, and the source of heterogeneity could not be identified. (4) Short-term follow-up might have led to an underestimation of complications.

## 5 Conclusion

The administration of TXA in primary SA could reduce postoperative hemoglobin reduction, total blood loss, and drainage volume and does not increase the risk incidence of complications. Due to the lack of sufficient high-quality evidence currently available, there is a need for additional large randomized controlled trials.
